# Factors Associated with Dietary Patterns in Colombia

**DOI:** 10.3390/nu15092079

**Published:** 2023-04-26

**Authors:** Luz Adriana Meneses-Urrea, Manuel Vaquero-Abellán, Dolly Villegas Arenas, Narly Benachi Sandoval, Mauricio Hernández-Carrillo, Guillermo Molina-Recio

**Affiliations:** 1 Research Group “Health Care (Recognized by Colciencias)”, University Santiago of Cali, Cali 760001, Colombia; dolly.villegas00@usc.edu.co (D.V.A.); nbenachi@clinic.cat (N.B.S.); 2 Department of Nursing, University Santiago of Cali, Cali 760001, Colombia; 3 IMIBIC GC12 Clinical and Epidemiological Research in Primary Care (GICEAP), 14014 Córdoba, Spain; mvaquero@uco.es; 4 Department of Nursing, Pharmacology and Physiotherapy, University of Córdoba, 14014 Córdoba, Spain; 5 CAP Casanova, Consorci D’Atenció Primària de Salut Barcelona Esquerra, 08036 Barcelona, Spain; 6 Department of Public Health, Mental Health and Maternal and Child Health Nursing, University of Bacelona, 08036 Barcelona, Spain; 7 Facultad de Salud, Universtiy of the Valle, Cali 760001, Colombia; mauriciohc@gmail.com; 8 Lifestyles, Innovation and Health (GA-16), Maimonides Biomedical Research Institute of Córdoba (IMIBIC), 14014 Córdoba, Spain

**Keywords:** food consumption, socioeconomic factors, spatial analysis

## Abstract

The selection of food depends on various factors such as cultural, social, economic and biological. This paper determines the factors associated with dietary patterns in Colombia. It is an observational, descriptive exploratory study collecting secondary data from the National Survey of Nutritional Status of Colombia (ENSIN, 2015) of 16,216 people between 15 and 64 years of age. The variables were the following: area, age range, sex, educational level, high blood pressure arterial hypertension (HTA), diabetes (DM), cancer, wealth quartile and dietary pattern. For the data analysis, logistic regression models were generated for each pattern and OR was used as a measure of association. Of those studied, 74.6% live in urban areas, all were aged between 15 and 49 years and 45.4% were in the first wealth quartile (Q1). There was a greater probability of traditional and conservative dietary patterns in people with diabetes and hypertension. Consumption of the conservative pattern was associated with being a woman, while consumption of the traditional pattern was associated with people in the first and second wealth level. Consumption of grill/beverage was more likely in men. Socio-demographic factors and chronic non-communicable diseases are associated with dietary patterns. This makes it relevant for health professionals to take into account these characteristics for nutritional interventions.

## 1. Introduction

Eating patterns are given by the consumption of those foods that are ingested more frequently and that exert synergistic effects on the body to meet physiological needs. In addition, they are an important component in understanding the relationship between food systems and food and nutrition security [1]. 

Nutritional epidemiological studies have focused on the analysis of the pattern that describes the general diet of a population, in order to understand the relationship between this and health status, which should guide the development of food policies. That is, the effectiveness of food policies and strategies depend on the knowledge and contextualization of the behavior of the population’s eating behavior, its culture and other factors that may influence the modification of lifestyles, which are capable of influencing the adoption of new eating patterns [2].

In this sense, the eating behavior of the population is influenced by the economic and structural development of society. In Western and high-income countries, the pattern is usually marked by a high consumption of fats, mainly saturated, sugars, processed foods and animal proteins, and a low intake of fiber and complex carbohydrates [3].

On the other hand, in the countries of Latin America and the Caribbean, the United Nations Organization reports that dietary patterns have traditionally been marked by a set of staple foods formed by cereals, roots and tubers. It is estimated that 39% of the dietary energy available in the countries of the region comes from these foods. For the Caribbean and South America it is 37%, while in Mesoamerica it accounts for 44% of the daily kilocalories available [1].

The origin of the kilocalories consumed has changed, causing a change in dietary patterns and an increase in the availability of proteins of animal origin, as well as animal and vegetable fats [4].

In Colombia, Meneses et al., through data obtained from the National Survey of the Nutritional Situation of Colombia (ENSIN) of 2015, with a population aged 15 to 64, identified four food patterns. The first, called “traditional pattern”, was made up of the food groups: dairy, potatoes/legumes, cereals, fried foods, coffee, panela/sugar/honey and meat/fish/eggs/sausages. The second food pattern, “industrial pattern”, included packaged treats, fast food and soft drinks. The third, called “conservative”, was made up of whole foods, light foods/supplements, fruits and vegetables. Finally, the fourth, “grilled food/processed drinks pattern”, was made up of alcoholic beverages, grill foods and energy drinks [5].

Therefore, we took as a reference these four patterns (traditional, industrialized, preservative, grilled food/processed drinks) and the influence of factors such as age, sex, socioeconomic level, schooling, ethnicity, etc. in feeding practices [6,7]. The research aimed to determine the factors associated with these patterns and their spatial distribution by the departments of Colombia. 

## 2. Methodology

This is an observational, descriptive exploratory study that uses secondary data from the sample of 16,216 people, between 15 and 64 years old, who participated in the National Survey of the Nutritional Situation of Colombia (ENSIN, 2015), a cross-sectional population study with probabilistic, cluster, stratified and multistage sampling [8].

The independent variables were area, age range, sex, education level, hypertension (HTN), diabetes (DM), cancer and quartile of wealth. The dependent variable was “dietary patterns”, defined according to the results obtained by this group and previously [5,7]. In this research, the authors defined the four food patterns discussed above: traditional pattern, industrial pattern, the preservative and the grill/beverage.

To determine the factors associated with dietary patterns and their spatial distribution in the Departments of Colombia, it was necessary to generate a new dichotomous dependent variable, through a binomial distribution, as follows: the cases that presented some degree of consumption of the corresponding pattern were categorized as 1, while those that did not reported a level of consumption of the pattern was categorized as 0. This was necessary to perform non-conditional logistic regression models [7].

These were elaborated, including all the available variables, that is, explanatory and adjustment, which were measured in a polytomous way (area, age ranges, educational level, quartile of wealth) and dichotomous (sex, hypertension, diabetes, cancer). From them, the coefficients associated with each variable were obtained, and the adjusted odds ratio (OR), where the reference category of each independent variable was the first.

The authors used backward modelling (as usual in this research type [9]). This modelling consists of starting with a saturated model that includes as many explanatory variables as possible (a *p*-value < 0.2 was set for the first modelling [9]) and then eliminating in consecutive steps those with *p* > 0.05. That is, only the significant ones remain (*p* < 0.05).

The R2 of Cox and Snell, the Hosmer and Lemeshow test and the logarithm of the likelihood (LL) were used as measures of goodness of fit of the models to assess the association of these variables with the explanation of each of the dietary patterns, generating logistic regression models for each pattern and using the OR association measure with its corresponding 95% confidence interval. 

From the data, a description of the behavior of food consumption by department was made, elaborating a map of Colombia that described the consolidation of these in quartiles (Q), each one represented by color intensity. The most intense meant greater presentation of consumption of the pattern in the upper quartile. Qgis version 3.10.13 [10] and SPSS version 27 was used for statistical analysis.

### Ethical Considerations 

The study was endorsed by the Institutional Ethics Review Committee of the Santiago de Cali University Approval Act No. 11 of 29 May 2020. The database used was authorized by the Colombian Institute of Family Welfare (ICBF) to conduct the research.

## 3. Results 

Most of the participants resided in the city (74.6%) and the most frequent age range was 15 to 29 years (54.0%). The majority were women (57.0%), and, in terms of educational level, it prevailed between primary and secondary school (79.5%). A quarter (25.1%) had high blood pressure, 1.6% diabetes mellitus and less than 1% had a history of cancer. In relation to the quartile of wealth, 45.4% were located in the first quartile (Table 1).

**Figure 1:**
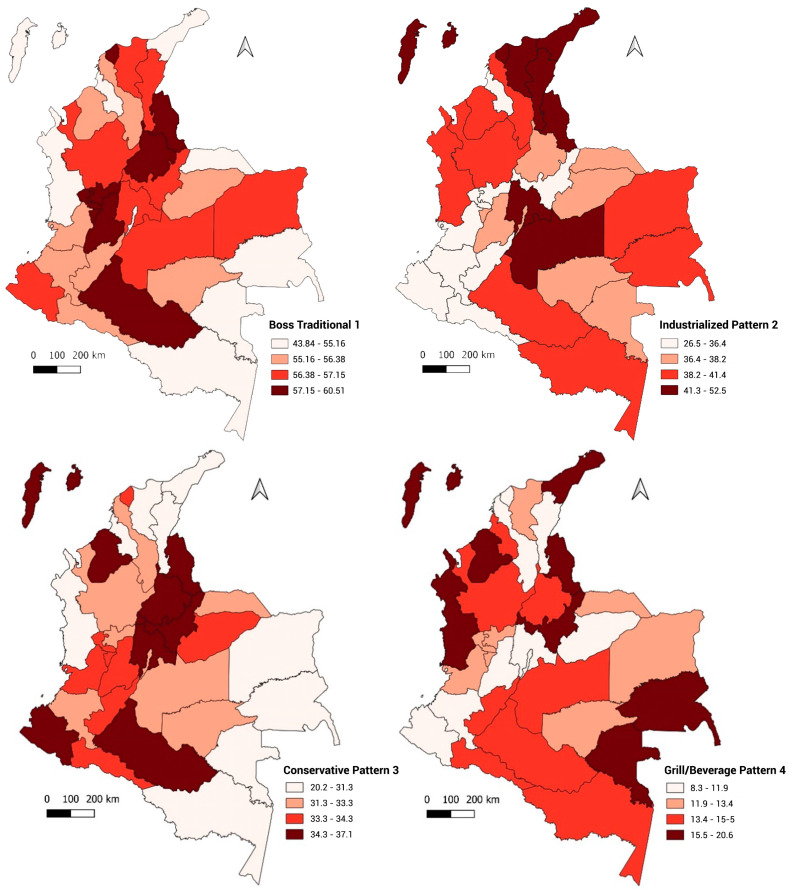
Map of the distribution of dietary patterns by department.

In the patterns of food consumption by department, the traditional pattern (pattern 1) had a higher consumption (between 57.1% and 60.5%) in the departments of Atlántico, Caldas, Tolima, Caquetá, Santander and Norte de Santander. The industrial pattern (pattern 2) was in the departments of La Guajira, Magdalena, Cesar, Cundinamarca, Meta, San Andrés and Providencia, with an average consumption between 41.3 and 52.5%. The conservative pattern (pattern 3) was more present in the departments of Córdoba, Santander, Norte de Santander, Cundinamarca, Boyacá, Caquetá, Nariño, San Andrés and Providencia, with percentages between 34.3% and 37.1%. The grill/beverage (pattern 4) stood out in the departments of La Guajira, Córdoba, Choco, Norte de Santander, Boyacá, Casanare, Guainía and Mitú, with the average consumption between 15.5% and 20.65% (Figure 1).

A modelling process was carried out for each dietary pattern. It started with a saturated model including eight explanatory variables: area, age range, sex, education level, hypertension (HTN), diabetes mellitus (DM), cancer and quartile of wealth. After the modeling process that began with the eight variables discussed above, four dietary patterns were identified (Table 2). Pattern 1, called “Boss Traditional”, was defined by six variables, highlighting its relationship with HTN and diabetes. Pattern 2, named “Industralized”, included six variables prominently related to different educational levels, wealth quartiles and HTN. In addition, pattern 3, which included five variables, was called “Conservative Pattern”, highlighting among them HTN, cancer and diabetes, which led us to believe that it was a dietary pattern more common in people with a chronic non-communicable disease and in which adherence to a healthy diet is a fundamental part of the treatment. Finally, pattern 4, called “Grill/Beverage”, maintained the eight initial variables of the saturated model, emphasizing its relationship with cancer, diabetes and living in a rural area. Possibly, this pattern represents subjects whose lifestyles (especially dietary habits) are related to the origin of these chronic pathologies; however, unlike the individuals most characterized by pattern 3, perhaps they have not abandoned these unhealthy dietary habits, which possibly played a role in the development of these diseases. More details about the patterns, the variables included in each one, and their degree of significance are available in Table 2 and Table 3.

**Figure 2:**
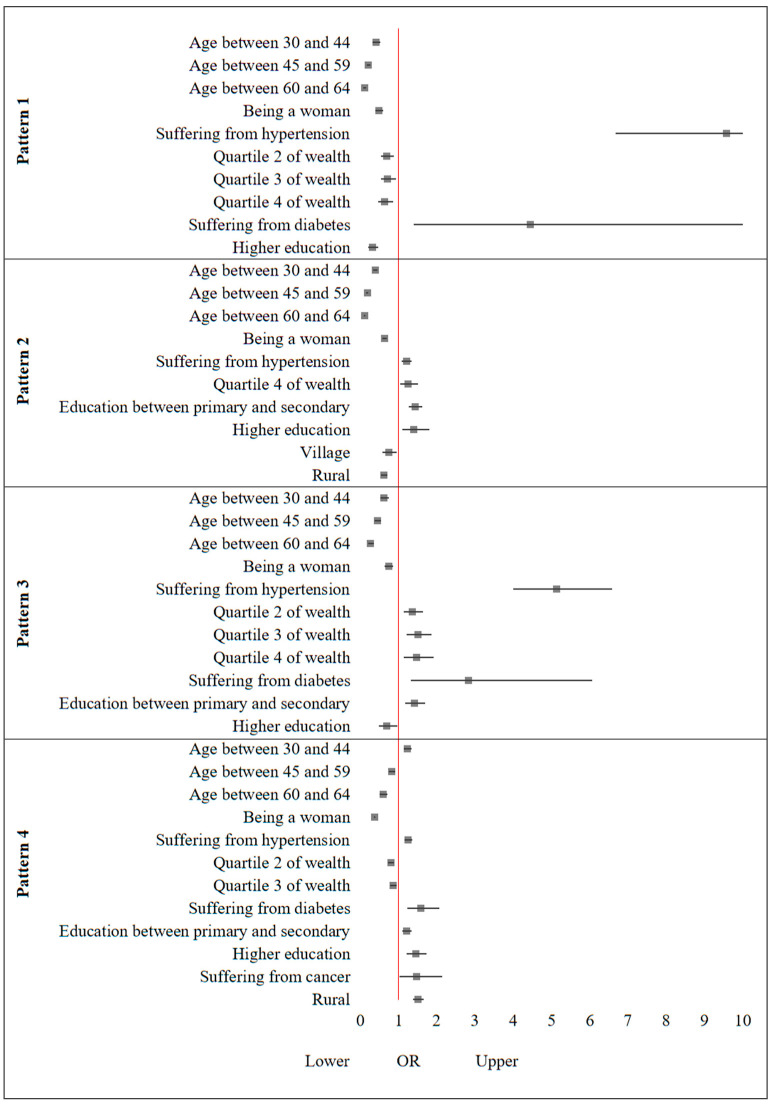
Confidence intervals (95%) of the odds ratios adjusted for the models obtained according to consumption pattern in Colombia.

Figure 2 shows the graph of the OR and confidence intervals adjusted by the dietary pattern, where the relevance of suffering from a chronic disease in relation to adopting pattern 1 and 3, the positive relationship between socioeconomic and cultural characteristics with patterns 2 and 3 or the role of the youngest age group in relation to pattern 4 can be observed. 

## 4. Discussion

The findings demonstrated the association between each eating pattern with the following variables: age group, sex, quartile of wealth and educational level. Similarly, it was found that living in a certain population area of Colombia (city, village, rural) or suffering from hypertension, diabetes mellitus or cancer, were not determining variables in the construction of the associated factors of some dietary patterns.

The population between 15 and 29 years old had a greater tendency to consume foods included in the industrialized pattern (sweets/package foods, fast food and soft drinks), followed by the traditional pattern (dairy, potatoes/legumes, cereals, fried foods, coffee, panela/sugar/honey and meat/fish/eggs/sausages). Like us, the study reported by Khandpur et al., found a greater tendency in young people to consume foods included in this pattern, probably influenced by marketing and marketing practices of these products [11].

In the population between 30 and 64 years, the consumption of foods included in the grill/beverage (alcoholic beverages; grill foods; energy drinks) was significantly more frequent, followed by the conservative pattern (whole foods, light foods/supplements, fruits and vegetables). This was similar to that found by Adrogue et al., where the average consumption of fruits and vegetables increased from the age of 35 [12].

Men were most likely to consume the grill/beverage pattern, followed by the traditional pattern. Women tended to consume more foods from the conservative pattern, followed by the industrialized pattern. Along these lines, several authors have indicated that women have greater awareness and knowledge of nutrition than men, greater motivation for weight control and prioritize healthy eating [13,14]. On the other hand, studies report that the consumption of energy drinks is associated with masculinity and extreme sports [15,16].

Suffering from hypertension was associated with all dietary patterns, with a greater tendency to consume foods included in the traditional pattern, followed by the conservative pattern. On the other hand, suffering from diabetes mellitus was strongly associated with the traditional pattern, followed by the conservative pattern and grill/beverage, while suffering from cancer was strongly associated with the conservative pattern, followed by the grill/beverage. Contrary to what might be expected, the conservative pattern had a significant association with these diseases, which could be explained by the years of diagnostic evolution and the probable modification of eating habits to control the evolution of the disease. According to several studies, there is a positive relationship between the frequency of fruit and vegetable consumption in chronic disease [17,18].

Regarding the traditional pattern, our findings agree with other authors [19,20], mentioning that patients with hypertension and diabetes mellitus have a greater tendency to consume dairy products, potatoes/legumes, cereals, fried foods, coffee, panela/sugar/honey and meat/fish/eggs/sausages. These foods are part of the food culture of the Colombian population, with a high caloric intake and are easy to acquire, due to their wide availability and low economic cost [21,22]. On the other hand, it has been shown that the consumption of whole grains containing bioactive components such as dietary fiber, resistant starch, vitamins, minerals and phytoestrogens [23,24], and the consumption of fruits and vegetables, are related to the control and reduction of the risk of cancer, hypertension, diabetes mellitus, obesity and other cardiovascular diseases [25,26]. According to Afshin et al., the average consumption of fruits worldwide is two-thirds of the minimum recommended amounts [27]. These vary in Central Asia, North Africa and the Middle East, where slightly more than the recommended minimum is consumed, while sub-Saharan Africa and Oceania consume only a third of this amount. The inhabitants of the Caribbean consume the most fruits, while those of southern Africa are the least [28]. Therefore, increasing the consumption of fruits and vegetables has become a priority globally, which is why it has been linked to the UN Sustainable Development Goals for the year 2030 [28]. However, increasing the presence of this pattern entails great difficulty because it is determined both by individual factors and by factors of the social and economic structure, which in many cases cannot be controlled by the individual [29].

Regarding the wealth index, quartiles three and four were strongly associated with the conservative pattern, followed by the industrialized pattern [30,31]. Quartiles of wealth one and two had the greatest association with the traditional pattern, followed by the grill/beverage. According to Miramontes et al. [32] and in the analysis of ENSIN 2005, people with low quartiles of wealth more frequently consumed foods included in the traditional pattern, such as tubers and bananas [33]. These differences reflect the influence of purchasing power on accessibility to healthy and unhealthy foods, causing changes in purchasing patterns and resulting diets [4,34].

Regarding the level of education, the population between primary and secondary school had a greater tendency to consume foods of the industrialized pattern, followed by the conservative pattern and grill/beverage. The population with a higher education had a greater tendency to consume foods of the grill/beverage pattern, followed by the industrialized pattern. This coincides with Lacko et al. who reported that the lower the educational level, the greater the intake of foods with high sugar content and packaged foods [35].

Living in a village or rural population area was strongly related to the grill/beverage and living in the city with the industrialized pattern. A study in 18 low-, middle- and high-income countries in seven geographic regions revealed that the highest consumption of fruits and vegetables was found in the urban area or municipal capital [36], which coincides with the results of this research.

Given that health levels vary substantially between regions, it is necessary to characterize these regional variations in order to identify areas with an accumulation of health problems and to improve epidemiological research on which appropriate public health policy decision-making should be supported [37]. Recent technological advances, such as geographic information systems (GIS), have enabled the application not only of disease mapping but also of spatial analyses, such as spatial clustering and cluster detection, in epidemiological research [38]. In this context, clusters are defined as unusual concentrations of health events in both space and time [39] and knowing how they behave in these clusters allows targeted interventions.

From this perspective of spatial distribution, there is a tendency to present the influence of several eating patterns in the same department, probably due to internal migratory phenomena in Colombia [40,41,42].

Even so, in the departments of Atlántico, Caldas, Caquetá, Norte de Santander, Santander and Tolima, there was a greater tendency to the traditional pattern, which may be related to the fact that these, with the exception of Atlántico and Caquetá, concentrate the second largest food production of cereals, legumes, tubers and sugar cane in Colombia [43]. At the same time, this type of food consumption had a strong association with quartiles of wealth one/two, a level of education lower than complete primary and suffering from hypertension or diabetes mellitus. As indicated by other authors, inadequate eating practices are associated with low income and lack of adequate food plans according to their sociocultural context [20], in addition to little knowledge about eating patterns [44], which makes it difficult to control chronic diseases.

On the other hand, the departments of Boyacá, Caquetá, Córdoba, Cundinamarca, Nariño, Norte de Santander, Santander, San Andrés and Providencia, had a greater tendency to the conservative pattern and this type of food consumption had a strong association with the quartiles of wealth three/four, educational level between primary and secondary, being a woman and suffering from cancer. According to the report of the National Administrative Department of Statistics (DANE) of Colombia, the departments of Boyacá, Nariño, Risaralda, Tolima and Valle del Cauca represent 53.7% of the production of vegetables in the rural area surveyed [45], coinciding with the results obtained in the spatial distribution of the pattern that includes a high consumption of the same (conservative).

Among the limitations of the study, underreporting in the comorbidity declaration is considered, given that the figures obtained do not correspond to what was expected [46]. As it is a study carried out from a secondary source of data, it was not possible to include cultural variables that could be influencing food consumption habits, in addition to variables related to access to healthy foods, years of diagnostic evolution and modification of eating habits to control the evolution of diseases: high blood pressure, diabetes mellitus and cancer. In addition, for future studies, it is recommended that analyses be carried out by subgroups and by specific territories, so that the results of the analyses can be used to evaluate health status and thus guide the development of food policies in a more focused manner. It would be important to be able to compare the dependence of the presence of hypertension on dietary patterns in each age group and in relation to the territory, given that hypertension is an age-related disease with a multifactorial etiology.

## 5. Conclusions

The study’s findings show that in Colombia, four dietary patterns are consumed in different proportions in each region, probably conditioned by migratory movements and the diversity of foods in the country. In contrast, dietary patterns were influenced by educational level, gender, chronic disease and economic and social conditions rather than by residence in a given area. We consider this information relevant for conducting epidemiological studies that allow them to relate to chronic non-communicable diseases and plan population interventions by regions to promote home practices and healthy habits that reduce their incidence.

## Figures and Tables

**Table 1:** Description of the variables included in the study, represented in absolute and relative frequencies.

Variable	Category	*n* = 16.216	%
Area	City	12.099	74.6%
	Village	583	3.6%
	Rural	3.534	21.8%
Age ranges	15–29 years	8.753	54.0%
	30–44 years	3.596	22.2%
	45–59 years	3.114	19.2%
	60–64 years	753	4.6%
Sex	Man	6.969	43.0%
	Woman	9.247	57.0%
Educational level *	Less than primary (0–4 years)	2.464	15.2%
	Education between primary and secondary	12.893	79.5%
	Higher education	778	4.8%
Hypertension	No	12.142	74.9%
	Yes	4.074	25.1%
Diabetes	No	15.951	98.4%
	Yes	265	1.6%
Cancer	No	16.084	99.2%
	Yes	132	0.8%
Quartile of wealth	First quartile	7.360	45.4%
	Second quartile	3.886	24.0%
	Third quartile	3.046	18.8%
	Fourth quartile	1.924	11.9%

* 81 without educational level registration.

**Table 2:** Comparison of logistic regression models for each dietary pattern with their respective measures of goodness of fit. Colombia.

Dietary Pattern	Model	# Var	# Sig Var	LL-2	R^2^ of Cox and Snell	R^2^ of Nagelkerke	Chi^2^ *	*p*-Value
Boss Traditional (1)	1	8	5	3743.23	0.028	0.1214	33.42	<0.001
	2	7	5	3743.30	0.028	0.1213	32.89	<0.001
	**3**	**6**	**6**	**3752.03**	**0.027**	**0.119**	**32.85**	**<0.001**
Industrialized Pattern (2)	1	8	6	11,168.16	0.084	0.155	18.850	0.016
	2	7	6	11,168.18	0.084	0.155	17.685	0.024
	**3**	**6**	**6**	**11,168.20**	**0.084**	**0.155**	**17.858**	**0.022**
Conservative Pattern (3)	1	8	6	6313.00	0.025	0.075	26.476	0.001
	**2**	**6**	**5**	**6315.15**	**0.025**	**0.074**	**34.853**	**<0.001**
Grill/Beverage (4)	**1**	**8**	**8**	**21,104.06**	**0.071**	**0.096**	**12.996**	**0.112**

* Hosmer and Lemeshow test; Model: order number of the model in the modelling sequence (the last one represents the final model). # Var: number of variables in the initial saturated model, # sig var: number of significant variables, Sig: significant, LL: logarithm of the likelihood. Models adjusted for the following: area, age group, sex, educational level, hypertension, diabetes, cancer and quartile of wealth.

**Table 3:** Adjusted association measures obtained from the definitive logistic regression model for each dietary pattern. Colombia.

Dietary Pattern	Variable	OR	95% C.I. Para OR		*p*-Value
			Lower	Upper	
Boss Traditional (1)	Age between 30 and 44	0.410	0.317	0.532	0.000
	Age between 45 and 59	0.208	0.162	0.268	0.000
	Age between 60 and 64	0.106	0.075	0.150	0.000
	Being a woman	0.479	0.390	0.590	0.000
	Suffering from hypertension	9.579	6.687	13,720	0.000
	Quartile 2 of wealth	0.693	0.542	0.886	0.003
	Quartile 3 of wealth	0.713	0.546	0.930	0.013
	Quartile 4 of wealth	0.631	0.466	0.855	0.003
	Suffering from diabetes	4.448	1.406	14,070	0.011
	Between primary and secondary education	0.784	0.595	1.033	0.084
	Higher education	0.316	0.210	0.475	0.000
Industrialized Pattern (2)	Age between 30 and 44	0.385	0.337	0.441	0.000
	Age between 45 and 59	0.183	0.160	0.209	0.000
	Age between 60 and 64	0.109	0.090	0.131	0.000
	Being a woman	0.632	0.571	0.699	0.000
	Suffering from hypertension	1.202	1.074	1.346	0.001
	Quartile 2 of wealth	1.054	0.915	1.214	0.463
	Quartile 3 of wealth	1.168	0.997	1.369	0.055
	Quartile 4 of wealth	1.253	1.041	1.507	0.017
	Education between primary and secondary	1.437	1.270	1.626	0.000
	Higher education	1.406	1.100	1.797	0.007
	Village	0.742	0.578	0.954	0.020
	Rural	0.620	0.543	0.709	0.000
Conservative Pattern (3)	Age between 30 and 44	0.625	0.518	0.753	0.000
	Age between 45 and 59	0.443	0.365	0.538	0.000
	Age between 60 and 64	0.267	0.202	0.353	0.000
	Being a woman	0.743	0.644	0.857	0.000
	Suffering from hypertension	5.131	4.000	6.582	0.000
	Quartile 2 of wealth	1.359	1.130	1.634	0.001
	Quartile 3 of wealth	1.503	1.216	1.858	0.000
	Quartile 4 of wealth	1.480	1.141	1.920	0.003
	Suffering from diabetes	2.833	1.324	6.060	0.007
	Education between primary and secondary	1.410	1.172	1.696	0.000
	Higher education	0.691	0.492	0.970	0.033
	Suffering from cancer	3.932	0.962	16,071	0.057
Grill/Beverage (4)	Age between 30 and 44	1.232	1.131	1.343	0.000
	Age between 45 and 59	0.829	0.754	0.911	0.000
	Age between 60 and 64	0.597	0.506	0.704	0.000
	Being a woman	0.372	0.348	0.397	0.000
	Suffering from hypertension	1.247	1.149	1.354	0.000
	Quartile 2 of wealth	0.802	0.732	0.878	0.000
	Quartile 3 of wealth	0.865	0.783	0.955	0.004
	Quartile 4 of wealth	0.980	0.872	1.101	0.737
	Suffering from diabetes	1.589	1.224	2.061	0.000
	Education between primary and secondary	1.207	1.092	1.334	0.000
	Higher education	1.452	1.215	1.736	0.000
	Suffering from cancer	1.479	1.025	2.134	0.037
	Village	1.046	0.876	1.248	0.620
	Rural	1.508	1.371	1.659	0.000

## Data Availability

The data will be made available to anyone who requests it from the corresponding author through a reasoned request.
